# Gender Incongruence of Adolescence and Adulthood: Acceptability and Clinical Utility of the World Health Organization’s Proposed ICD-11 Criteria

**DOI:** 10.1371/journal.pone.0160066

**Published:** 2016-10-24

**Authors:** Titia F. Beek, Peggy T. Cohen-Kettenis, Walter P. Bouman, Annelou L. C. de Vries, Thomas D. Steensma, Gemma L. Witcomb, Jon Arcelus, Christina Richards, Els Elaut, Baudewijntje P. C. Kreukels

**Affiliations:** 1 Department of Medical Psychology & Center of Expertise on Gender Dysphoria, VU University Medical Center, Amsterdam, The Netherlands; 2 Nottingham Centre for Gender Dysphoria, Nottingham, United Kingdom; 3 School of Sport, Exercise, and Health Sciences, Loughborough University, Loughborough, United Kingdom; 4 Division of Psychiatry and Applied Psychology, Faculty of Medicine & Health Sciences, University of Nottingham, Nottingham, United Kingdom; 5 Center for Sexology and Gender Problems, Ghent University Hospital, Ghent, Belgium; University of Toronto Dalla Lana School of Public Health, CANADA

## Abstract

The World Health Organization (WHO) is currently updating the tenth version of their diagnostic tool, the International Classification of Diseases (ICD, WHO, 1992). Changes have been proposed for the diagnosis of Transsexualism (ICD-10) with regard to terminology, placement and content. The aim of this study was to gather the opinions of transgender individuals (and their relatives/partners) and clinicians in the Netherlands, Flanders (Belgium) and the United Kingdom regarding the proposed changes and the clinical applicability and utility of the ICD-11 criteria of ‘Gender Incongruence of Adolescence and Adulthood’ (GIAA). A total of 628 participants were included in the study: 284 from the Netherlands (45.2%), 8 from Flanders (Belgium) (1.3%), and 336 (53.5%) from the UK. Most participants were transgender people (or their partners/relatives) (*n* = 522), 89 participants were healthcare providers (HCPs) and 17 were both healthcare providers and (partners/relatives of) transgender people. Participants completed an online survey developed for this study. Most participants were in favor of the proposed diagnostic term of ‘Gender Incongruence’ and thought that this was an improvement on the ICD-10 diagnostic term of ‘Transsexualism’. Placement in a separate chapter dealing with Sexual- and Gender-related Health or as a Z-code was preferred by many and only a small number of participants stated that this diagnosis should be excluded from the ICD-11. In the UK, most transgender participants thought there should be a diagnosis related to being trans. However, if it were to be removed from the chapter on “psychiatric disorders”, many transgender respondents indicated that they would prefer it to be removed from the ICD in its entirety. There were no large differences between the responses of the transgender participants (or their partners and relatives) and HCPs. HCPs were generally positive about the GIAA diagnosis; most thought the diagnosis was clearly defined and easy to use in their practice or work. The duration of gender incongruence (several months) was seen by many as too short and required a clearer definition. If the new diagnostic term of GIAA is retained, it should not be stigmatizing to individuals. Moving this diagnosis away from the mental and behavioral chapter was generally supported. Access to healthcare was one area where retaining a diagnosis seemed to be of benefit.

## Introduction

The World Health Organization (WHO) is currently updating the tenth edition of their diagnostic tool; the International Classification of Diseases (ICD [1]). The ICD is used as a diagnostic tool for epidemiology, health management and clinical purposes and aids the monitoring of the incidence and prevalence of diseases and other health problems in different countries and populations. The ICD-11 is scheduled to be released in 2018 [2], making the 26 year period between both editions without a revision the longest in the ICD’s history.

As part of the development of the new edition of the ICD the diagnostic category of “Gender Identity Disorders” (F64), which includes Transsexualism (F64.0); Dual-role Transvestism (F64.1); Gender Identity Disorder of Childhood (F64.2); Other Gender Identity Disorders (F64.3); and Gender Identity Disorder, Unspecified (F64.4) is being reviewed. WHO’s Working Group on the Classification of Sexual Disorders and Sexual Health (WGSDSH) was given the task of “reviewing and evaluating clinical and research data informing gender identity diagnoses since the publication of the ICD-10 in 1992” [3]. Based on their review, the WGSDSH wrote:

“…it is now appropriate to abandon the psychopathological model of transgender people based on 1940s conceptualizations of sexual deviance and to move towards a model that is (1) more reflective of current scientific evidence and best practices; (2) more responsive to the needs, experience, and human rights of this vulnerable population; and (3) more supportive of the provision of accessible and high-quality healthcare services”[3].

The revision of the diagnosis related to gender identity was expected to spark debates, as has been the case when developing the new diagnosis of Gender Dysphoria as part of the fifth edition of the Diagnostic and Statistical Manual of Mental Disorders (DSM) from the American Psychiatric Association (APA) (e.g. [3–7]). The WHO has received many proposals calling for de-pathologization of a diagnosis of *gender identity disorder* and its removal from the mental disorders section of the classification (e.g. from Global Action for Trans* Equality–GATE [8] and the International Campaign Stop Trans Pathologisation [9]). Furthermore, the European Parliament called on the Commission and the World Health Organization to withdraw *gender identity disorders* from the mental and behavioral disorders chapter, and to ensure a non-pathologising reclassification in the negotiations on the ICD-11 [10]. The desire to destigmatize gender incongruence and the importance of securing access to care has been described as the central dilemma in both the DSM-5 and ICD-11 revision processes [3].

The WGSDSH has proposed to change the name of the diagnosis from *Transsexualism/Gender Identity Disorder* to *Gender Incongruence* and to remove it from the chapter of Mental and Behavioral Disorders to a new chapter (*Conditions Related to Sexual and Gender Health)* [3]. Currently, four diagnoses have been proposed within the diagnostic category of *Gender Incongruence*, namely Gender Incongruence of adolescence and adulthood (5A30); Gender Incongruence of Childhood (5A31); Other Specified Gender Incongruence (5A3Y) and Gender Incongruence, Unspecified (5A3Z). See [11].

Some of the suggested changes are:

1. Distress or impairment of functioning not to be required for the diagnosis.
2. Gender Incongruence to be present for a shorter period of time: several months as opposed to two years as per the ICD-10.
3. The wording used to be less binary and therefore the diagnosis to be applicable to a larger group of individuals. The term “experienced gender” to be used as opposed to “opposite sex” or “preferred sex” as currently used in the ICD-10. (See S1 Text for the final draft criteria of ‘Gender Incongruence of Adolescence and Adulthood’ of the WGSDSH criteria used in this study).

In the proposed diagnosis, the person will not be required to experience *serious distress* or *functional impairment* (e.g. inability to function properly in their job or social life) as a consequence of their gender incongruence in order to fulfill the diagnostic criteria. Formerly, people would *only* be eligible for the diagnosis, and therefore treatment [3,4], if they experienced serious distress from their gender incongruence or were unable to function properly because of it. Since most health insurance systems use a classification code as a requirement to offer healthcare, it is only when a person has a diagnosis that treatment can be provided. The proposed change entails that people who experience discomfort with their birth assigned gender but who do not experience significant distress or impaired ability to live a functional life (work, socialize, etc.) will now meet the diagnostic criteria and as result, be entitled to access to treatment in many countries. As a result of these broadened criteria, the diagnosis will be applicable to a more diverse group of individuals. Although this may result in increased accessibility to healthcare for transgender people wanting treatment, it may also pathologise individuals who might not have any problems with their gender incongruence nor desire any treatment. Fulfilling criteria for a diagnosis may be experienced by some as stigmatizing although the WGSDSH felt, that by removing it from the mental disorder chapter stigmatization should be reduced [3].

The WHO encouraged the public to provide feedback on the proposed ICD-11 and made the proposed criteria available online [11]. Further input is expected through the results of several field trials in lower and middle-income countries, funded by the WHO. As a part of this process and considering that the proposed changes have a number of advantages and disadvantages, the aim of our study was to gather the views of transgender individuals (and their relatives/partners) and clinicians in the Netherlands, Flanders (Belgium) and the United Kingdom regarding the proposed changes and the applicability and utility of the diagnostic criteria of *Gender Incongruence of Adolescence and Adulthood* for clinicians. The opinions regarding the GIC diagnosis will be reported elsewhere, see [12]. These countries were selected for a number of reasons. First, the Netherlands, Belgium, and the United Kingdom have established and well developed gender identity clinic services which broadly follow similar standards of care [13,14]. Secondly, they have a different healthcare system as The Netherlands and Flanders (Belgium) have a health insurance system whereas the United Kingdom has not. The study also aims to compare the responses between both countries.

## Method and Materials

### Method

#### Participants & procedure in the Netherlands and Flanders (Belgium)

Participants in this study consisted of healthcare providers as well as service users of transgender healthcare and other stakeholders (i.e., parents of trans children, and siblings or partners of trans people). Only people over the age of 16 years were included. The following procedure was followed for the recruitment of healthcare providers: Mental health professionals working in the field of gender incongruence were recruited from the VU University Medical Center Amsterdam Center of Expertise on Gender Dysphoria (VUmc-CEGD); the Gender Team of the University Medical Center Groningen and Transvisie zorg (an organization that provides information, counselling and psychological care for transgender people and their social environment) in The Netherlands; and the Center of Sexology and Gender of the University Hospital in Ghent, Belgium. Specialized healthcare providers were sent a link to an online survey to complete. Healthcare providers who were *not* specialized in transgender care (e.g., healthcare psychologists, psychiatrists, general practitioners, general practitioners trainees, social workers) were contacted via e-mail through the researchers’ networks and were–after agreeing to participate—invited via e-mail to the online survey. Furthermore, general psychiatrists and psychiatric trainees were invited to participate in the survey following a research session organized by the Dutch researchers. Some people completed the online version, while others completed a pen and paper version of the survey.

For the recruitment of service users, transgender adults who came to the VUmc- CEGD for an appointment were invited to participate in the study. If they were interested in participating, their e-mail address was passed to the researcher and a link to the survey was sent. Reasons as to why transgender adults refused to participate were also collected. Parents of children with gender dysphoria were approached using a mailing list from Dutch a support group for parents of transgender children. Parents attending the CEGD at the VUmc were also invited to participate.

A total of 758 people were invited to participate in the Dutch survey (from the Netherlands and Belgium). Of this group, 36 people refused participation for various reasons (multiple response options were possible): They did not feel like participating (*n* = 16); they did not have time (*n* = 11); it did not matter to them whether or not the diagnosis (terminology and criteria) was changed (*n* = 8); and other reasons (*n* = 5). Therefore, 722 agreed to participate and received a link to the survey via e-mail. Despite several reminders, 383 people (53.0%) failed to open the link to start the survey. At the start of the survey, participants first received information about the study and were then asked to give written consent by selecting “yes” in response to the question: “Do you agree to participate in this study?”. Forty-one people gave consent but did not answer any questions so they were removed from the data set. Another 6 participants were excluded as they answered fewer than 5 questions. A total of 292 Dutch-speaking participants (284 from the Netherlands, 8 from Belgium) started the survey and answered at least 5 questions (40.4% of those who received a link to the survey and 38.5% of all participants who were approached). All participants from Belgium were specialized Healthcare providers. The survey was not presented to transgender people/or their partners or relatives in Belgium. The survey was completed by 223 people (76.4% of included Dutch participants).

#### Participants & procedure in the United Kingdom

Information about the study (including the service user recruitment procedure) and information sheets were sent by the research team at the Nottingham Centre for Gender Dysphoria (NCGD) to every Gender Identity Clinic Service in the UK (*n* = 8) and the four main surgical centers specialized in trans surgery. Healthcare professionals working in these clinics were invited to participate, and if they agreed a link to the online survey was sent. Information regarding reasons for not participating were recorded. Service users were recruited from the nine Gender Identity Clinic Services, including the NCGD. Clinicians provided a brief explanation of the study to the service users and invited them to participate in the study by providing them with a very brief information sheet where a link to the survey could be found. Further information about the study and a consent form were hosted online. A paper copy was offered to potential participants who were not computer literate, had no access to online facilities, or preferred paper and pen. Only three individuals requested a paper copy and these were mailed out with a consent form, information sheet, and return stamped and addressed envelope; however none were returned. A snowballing method was used in order to increase recruitment, with service users sharing the online link with friends and family, and clinicians with their gender and non-gender specialist colleagues.

Due to the snowballing methods used the number of invitations sent is unknown. Overall, 552 UK participants entered the survey. Five people opted to answer the questionnaire regarding non-participation. The reasons given were: no opinion of whether the diagnosis changes (*n* = 1); technical issues (*n* = 1); not feeling knowledgeable enough (*n* = 1); adverse to the concept of diagnosis (*n* = 1); and concerns regarding online surveys (*n* = 1). In total, 387 completed the survey (70%) and only their responses were saved by the survey software. Seven participants did not progress past the information page, but all others continued to give consent. Participants then dropped out at various stages, the greatest number of drop-outs (*n* = 71) occurred for questions regarding the positioning of the diagnosis. Of the 387 participants who completed the survey, 50 were not from the United Kingdom and were excluded. One other person was excluded because they were under the age of 16. This resulted in a final UK sample of 336 participants.

#### Participants overall

A total of 628 participants were included in data analysis: 284 from the Netherlands (45.2%); 8 from Flanders (Belgium) (1.3%); and 336 (53.5%) from the UK (see Table 1 below).

**Table 1:** Frequency Table of Gender Assigned at Birth, Gender Identity, and Level of Education (and Percentages for each Column).

Demographic			Respondent Category										
			All		(Relatives/Partners of) Transgender People			Healthcare providers			Both healthcare providers and (Relatives/Partners of) Transgender People		
			Country of data collection		Country of data collection			Country of data collection			Country of data collection		
		Total (*n* = 628)	NL *(n* = 292)	UK (*n* = 336)	NL (*n* = 210)	UK (*n* = 312)	Total (*n* = 522)	NL (*n* = 72)	UK (*n* = 17)	Total (*n* = 89)	NL (*n* = 10)	UK (*n* = 7)	Total (*n* = 17)
Assigned gender	Male	334 (53.2%)	123 (42.1%)	211 (62.8%)	103 (49.0%)	195 (62.5%)	298 (57.1%)	17 (23.6%)	13 (76.5%)	30 (33.7%)	3 (30.0%)	3 (42.9%)	6 (35.3%)
	Female	292 (46.5%)	169 (57.9%)	123 (36.6%)	107 (51.0%)	115 (36.9%)	222 (42.5%)	55 (76.4%)	4 (23.5%)	59 (66.3%)	7 (70.0%)	4 (57.1%)	11 (64.7%)
	Neither	2 (0.3%)	0 (0.0%)	2 (0.6%)	0 (0%)	2 (0.6%)	2 (0.4%)	0 (0%)	0 (0%)	0 (0%)	0 (0%)	0 (0%)	0 (0%)
Gender Identity	Male	187 (29.8%)	104 (35.6%)	83 (24.7%)	81 (38.6%)	67 (21.5%)	148 (28.4%)	17 (23.6%)	13 (76.5%)	30 (33.7%)	6 (60.0%)	3 (42.9%)	9 (52.9%)
	Female	346 (55.1%)	163 (55.8%)	183 (54.5%)	105 (50.0%)	177 (56.7%)	282 (54.0%)	55 (76.4%)	4 (23.5%)	59 (66.3%)	3 (30.0%)	2 (28.6%)	5 (29.4%)
	Partly male, partly female	27 (4.3%)	10 (3.4%)	17 (5.1%)	9 (4.3%)	17 (5.4%)	26 (5.0%)	0 (0%)	0 (0%)	0 (0%)	1 (10.0%)	0 (0%)	1 (5.9%)
	Neither male nor female	26 (4.1%)	4 (1.4%)	22 (6.5%)	4 (1.9%)	22 (7.1%)	26 (5.0%)	0 (0%)	0 (0%)	0 (0%)	0 (0%)	0 (0%)	0 (0%)
	Don't know (yet)	5 (0.8%)	0 (0.0%)	5 (1.5%)	0 (0%)	5 (1.6%)	5 (1.0%)	0 (0%)	0 (0%)	0 (0%)	0 (0%)	0 (0%)	0 (0%)
	Other	37 (5.9%)	11 (3.8%)	26 (7.7%)	11 (5.2%)	24 (7.7%)	35 (6.7%)	0 (0%)	0 (0%)	0 (0%)	0 (0%)	2 (28.6%)	2 (11.8%)
Level of Education	Low	20 (3.2%)	15 (5.1%)	5 (1.5%)	15 (7.1%)	5 (1.6%)	20 (3.8%)	0 (0.0%)	0 (0.0%)	0 (0.0%)	0 (0.0%)	0 (0.0%)	0 (0.0%)
	Middle	251 (40.0%)	110 (37.7%)	141 (42.0%)	109 (51.9%)	141 (45.2%)	250 (47.9%)	0 (0.0%)	0 (0.0%)	0 (0.0%)	1 (10.0%)	0 (0.0%)	1 (5.9%)
	High	357 (56.8%)	167 (57.2%)	190 (56.5%)	86 (41.0%)	166 (53.2%)	252 (48.3%)	72 (100%)	17 (100%)	89 (100%)	9 (90.0%)	7 (100%)	16 (94.1%)

### Materials

The research team from the CEGD in Amsterdam developed a questionnaire with the input of transgender health care professionals and other stakeholders. This questionnaire covered questions regarding both the Gender Incongruence of Childhood classification (GIC) and the Gender Incongruence of Adolescence and Adulthood (GIAA The opinions regarding the GIC diagnosis will be reported elsewhere, see [12]. The final survey consisted of two parts (see S2 Text for the complete survey). The first part focused on the view of the service users and clinicians with regard to the various changes proposed in the new edition of the ICD (as compared to the ICD-10). The second part was only for healthcare providers and aimed to examine the clinical utility and the clinical implications of the proposed criteria. A special effort was made to explain concepts that some participants may have been unfamiliar with through the use of pop-up information windows that appeared when participants placed their mouse on the blue-colored, bold and underlined words. For example, explanations were provided on the concept of a Z-code (this concerns “factors that affect health and also influence contacts with the healthcare system” but does not concern diseases or disorders) and Disorders/Differences of Sex Development (DSD; when sex development is not completely in the typical male or female direction).

The Dutch survey went live on June 2^nd^ 2014 and was open for 10.5 months. The Dutch version of the questionnaire was translated into English by a Dutch translation company and then further amendments to the language were made by the research team in the UK (GW, WB & CR) to ensure appropriateness and ease of use by lay people. Following this, the questionnaire was sent to the lead clinicians at all Gender Identity Clinic services in the United Kingdom, as well as the main providers of gender-related surgery. A meeting took place where stakeholders could comment on the questionnaire and no major amendments were suggested, but the length of the questionnaire was questioned by various stakeholders. However, changes to this could not be made for reasons of consistency with the original Dutch version. During the same period, representatives from the major trans stakeholder groups in the United Kingdom were invited to meet and discuss the questionnaire in detail. The overarching remit given to this group was to remain as closely as possible to the original Dutch version of the questionnaire. Amendments were made to some of the language used, the order of questions, response options, and a small number of new questions was added. The final version was then translated back into Dutch by another Dutch translation company and shared with the team in the Netherlands. The online questionnaire went live in the UK on October 1^st^ 2014 and was open for 7.5 months.

The survey began with a series of demographic questions that asked about age, geographical location, gender identity (identification and description), birth assigned gender, education, employment status, and respondent category (i.e., trans, relative, healthcare provider). It then went on to ask about general opinions towards diagnosis and whether the respondent was receiving or had received treatment (if applicable). All questions were fixed-choice options, with spaces provided to answer “other” and add more information if required. The answer options going through the survey were a mixture of one-option responses or multi-option responses, depending upon the question. Options were provided to cover all eventualities, including neutral/no opinion; always with the addition of “other, please specify” or “please explain your answer” to allow for additional comments. For example, questions that asked for level of agreement would give seven main options: “Strongly agree, Agree, agree a little, Neutral; neither agree nor disagree, Disagree a little, Disagree, and Strongly disagree”, plus “please explain your answer”. Questions that asked about opinions (e.g., “Do you think….”) would give four main options: “Yes, No, No opinion, Don’t know” plus “please explain your answer”.

The Dutch study was granted full ethical approval (inclusion of Belgian participants and the consent method for participants under the age of 18 was covered) by the Commissie Wetenschappelijk Onderzoek [Committee of Scientific Research] of the EMGO Institute for Health and Care Research (EMGO^+^) with project ID WC2014-09 and the UK study was granted full Ethics Approval (the consent method for participants under the age of 18 was covered) by the UK National Research Ethics Service Committee East Midlands—Nottingham 1 with IRAS project ID 152591.

### Data analysis

Participants who were healthcare provider as well as a transgender person were included in both the Transgender group (TG) and the Healthcare providers (HCP) group. Differences between participants from the United Kingdom (UK) and participants from the Netherlands/Flanders (NL) were explored with a t-test for continuous data (age), Chi-square tests or Fisher’s Exact test (for the HCP only questions were the sample was smaller and Chi-square assumptions were violated) for categorical data and Mann-Whitney U tests for ordinal data or when t-test assumptions were violated. As participants who were both HCP and TG persons were included in both groups, the TG and HCP group were not independent. Therefore, no statistical differences between these groups were explored. The results of the HCP group are described after the TG group.

## Results

### Sample Characteristics

Based on the respondent type categories selected (see S3 Text for the additional information on respondent categories), participants were divided into three categories: healthcare providers (HCP), service users (transgender persons and their partners and/or relatives) (TG) and participants who are both healthcare providers and (partners/relatives of) transgender persons (Both). The TG group consists mainly of transgender persons (see Table A in S3 Text), but also parents of children/adolescents with gender incongruent feelings were included, as they have first-hand experience with the healthcare provided for their child. Furthermore, some partners and relatives of transgender persons were included in the TG group. The vast majority of all participants were service users (or their partners/relatives) (*n* = 522), 89 participants were healthcare providers and 17 participants were both healthcare providers and (partners/relatives of) transgender people, see Table 1. These three categories were used to analyse the responses on the survey questions.

The age of the participants ranged between 16 and 78 with a mean of 38.72 (*SD* = 14.59). There was no statistical difference between the mean age in the Netherlands (*M* = 38.47, *SD* = 13.64, *n* = 290) and the UK (*M* = 38.94, *SD* = 15.38, *n* = 336), *t*(624) = -.40, *p* = .69. The level of education did not differ between the Netherlands and the UK (*U* = 48589.5, *p* = .81). For sociodemographic characteristics on assigned gender, gender identity and level of education see Table 1.

For each question, the results of the transgender participants are described first, followed by the findings from the healthcare providers. Finally, the findings from the specific questions for healthcare providers are presented. Transgender participants who are healthcare providers are included in both the Transgender group and the Healthcare providers (HCP) group.

### General Features of the Diagnosis

#### Terminology

Respondents were asked what they thought of the statement that the ICD-10 diagnostic term of *transsexualism* should change on a seven point scale (from strongly agree to strongly disagree). In the TG group (*n* = 532), the mean agreement was 2.45 (*SD* = 1.62). When comparing the responses between the two countries a statistical difference was found (*U* = 28586, *z* = -3.23, *p* = .001, *r* = -.14). The UK TG participants scored lower (*M* = 2.32, *SD* = 1.65, *n* = 319, mean rank = 249.61) than the NL TG participants (*M* = 2.64, *SD* = 1.55, *n* = 213, mean rank = 291.79), indicating that the UK participants agreed stronger with the statement than the NL participants. When the possible responses were grouped into three categories (agree to various degrees, neutral, disagree to various degrees), it was found of the UK TG participants, 78.7% (*n* = 251) agreed with the statement compared to 67.6% (*n* = 144) of the NL TG participants. Of the NL participants, 22.5% (*n* = 48) reported being neutral compared to 11.6% (*n* = 37) of the UK participants. In NL 9.9% (*n* = 21) and in the UK 9.7% (*n* = 31) disagreed with the statement. Like the TG participants, the majority of HCPs (88.7%; *n* = 94) agreed (to various degrees) with the statement that the ICD-10 diagnostic term of *transsexualism* should change.

In the UK, most transgender participants considered the term *gender incongruence* to be an improvement on *transsexualism* (68.7%; *n* = 219). Most UK HCP also saw this as an improvement (83.3%, *n* = 20). Furthermore, *gender incongruence* was selected most frequently (22.2%; *n* = 70) as the preferred diagnostic term in the UK. Other frequently selected terms were: *gender dysphoria* (19.4%; *n* = 61), *gender variance* (13.3%; *n* = 42), *gender diversity* (11.7%; *n* = 37), or another term (12.1%; *n* = 38) (see Table 2). Examples of other terms include: “*Sex-based Dysphoria/ Dysmorphia*”; “*Misgendered at Birth*”; “*Gender fluidity*”; “*Transgender*” and “*Gender Condition*”. Rather than writing down a term, some participants gave statements, for example: “*Trans people should not be pathologised through medical diagnoses*.” or “*Culturally Induced Gender Anxiety Disorder”—caused by an inability to express our true selves due to social expectations*, *gender stereotypes*, *or situational constraints*.” In line with the UK TG respondents, most UK HCPs (54.2%, *n* = 13) preferred *gender incongruence* as a diagnostic term. Other terms selected were *gender diversity* (16.7%, *n* = 4), *gender variance* (12.5%, *n* = 3), *gender dysphoria* (12.5%, *n* = 3), or another term (4.2%, *n* = 1).

**Table 2:** Frequency Table (and Percentage of Column) of Responses to the Question (UK only): Do you consider the proposed name of gender incongruence an improvement on transsexualism?; and preferences for Diagnostic term.

Question	Possible answer	N and percentage response
Do you consider the proposed name of gender incongruence an improvement on transsexualism? (*n* = 319)	No	59 (18.5%)
	Yes	219 (68.7%)
	Doesn't matter	34 (10.7%)
	No opinion	7 (2.2%)
Preferences for Diagnostic Term (*n* = 315)	Gender transition	15 (4.8%)
	Gender diversity	37 (11.7%)
	Gender variance	42 (13.3%)
	Gender incongruence	70 (22.2%)
	Gender dysphoria	61 (19.4%)
	Trans	24 (7.6%)
	Transsexuality	12 (3.8%)
	Gender identity disorder	16 (5.1%)
	Other, please specify	38 (12.1%)

The Dutch survey was less specific in asking about the preferred diagnostic term. Dutch participants were asked their opinion regarding the following statement: “Do you consider the proposed name of gender incongruence an improvement on transsexualism or gender identity disorder?” Most transgender participants (and their family and/or partner) (58.0%; *n* = 120) responded with “yes” to this statement, 23.7% (*n* = 49) said ‘no’, 14.5% (*n* = 30) said “doesn’t matter” and 3.9% (*n* = 8) had no opinion. Most Dutch HCPs (79.7%, n = 63) also noted they saw Gender Incongruence as an improvement.

### Positioning of the Diagnosis

Respondents were asked what they thought of the statement that gender incongruence among adolescents/adults is a psychiatric disorder on a seven point scale (from strongly agree to strongly disagree). In the TG group (*n* = 524), the mean agreement was 5.73 (*SD* = 1.66). There were no differences between the countries, *U* = 35395.5, *z* = 1.70, *p* = .09, *r* = .07: the mean score of the UK TG participants (*n* = 319) was 5.85 (*SD* = 1.56, mean rank = 270.96) and 5.54 (*SD* = 1.79, mean rank = 249.34) of the NL TG participants (*n* = 205). When the possible responses were grouped into three categories (agree to various degrees, neutral, disagree to various degrees), it was found that 77.3% of the TG respondents (*n* = 405) disagreed (to various degrees) with the statement that “Gender incongruence in adolescents/adults is a psychiatric disorder”, 11.1% (*n* = 58) were neutral, and 11.6% (*n* = 61) agreed (to various degrees) with the statement. In line with the results of the transgender participants, the majority of the HCPs (57.3%; *n* = 59) disagreed (to various degrees) with the statement that gender incongruence among adolescents/adults is a psychiatric disorder, while 22.3% (n = 23) was neutral and 20.4% (n = 21) agreed.

When people were asked in which chapter they thought a diagnosis of gender incongruence for adolescents/adults should be included, a large majority of TG participants thought the diagnosis should be included somewhere in the ICD-11. However, 7.2% (*n* = 36) said that the diagnosis should not be in the ICD at all (see Table 3). Participants most frequently (38.1%; *n* = 191) reported thinking the gender incongruence diagnosis should be placed in a separate chapter dealing with symptoms and/or disorders regarding sexual- and gender-related health. The second most selected option was to include gender incongruence as a Z-code (26.5%; *n* = 133). There were statistical differences between the countries (*χ*2(8) = 38.54, *p* < .01, Cramer’s *V* = .28). In the UK, the most frequently selected option was to include a gender incongruence diagnosis as a Z-code (32.6%; *n* = 104) and the second most preferred option was a separate chapter dealing with symptoms and/or disorders regarding sexual and gender health (30.7%; *n* = 98). In contrast, most NL participants preferred gender incongruence to be placed in a separate chapter dealing with symptoms and/or disorders regarding sexual and gender health (51.1%; *n* = 93) and their next preferred placement was as a Z-code (15.9%; *n* = 29). Overall 49 HCPs (49.0%) thought the gender incongruence diagnosis should be placed in a separate chapter dealing with symptoms and/or disorders regarding sexual and gender health. The second most reported option was to include GIAA as a Z-code (21.0%; *n* = 21). Only 3 (3%) HCPs (1 from NL, 2 from the UK) thought gender incongruence should not be in the ICD at all. In line with the results of the Dutch TG participants, Dutch HCPs preferred the GIAA to be placed in a separated chapter (52.6%; *n* = 40) and the next preferred placement was as a Z-code (15.8%; *n* = 12). Similarly, in line with the UK TG paricipants, the most frequently selected placement options by UK HCPs was as a separate chapter (37.5%; *n* = 9) or as a Z-code (37.5%; *n* = 9).

**Table 3:** Frequency Table (and Percentage of Column) of Opinions Regarding the Statement: In what chapter of the ICD-11 do you think the diagnosis of gender incongruence for adolescents/adults should be included?

Chapter	Country of data collection		
	NL (n = 182)	UK (n = 319)	Total (n = 501)
Neurologic disorders and diseases	8 (4.4%)	14 (4.4%)	22 (4.4%)
Hormonal disorders and diseases	10 (5.5%)	19 (6.0%)	29 (5.8%)
Urogenital disorders and diseases	0 (0.0%)	3 (0.9%)	3 (0.6%)
Psychiatric disorders and diseases	8 (4.4%)	4 (1.3%)	12 (2.4%)
It should be part of several medical chapters simultaneously	11 (6.0%)	40 (12.5%)	51 (10.2%)
A separate chapter dealing with symptoms / disorders regarding sexual and gender health	93 (51.1%)	98 (30.7%)	191 (38.1%)
It should be a Z-code	29 (15.9%)	104 (32.6%)	133 (26.5%)
It should not be in the ICD at all	11 (6.0%)	25 (7.8%)	36 (7.2%)
Other	12 (6.6%)	12 (3.8%)	24 (4.8%)

The UK transgender participants (*n* = 319) were asked the three following questions (not present in the Dutch version). The first was: “Do you feel that there should be a diagnosis related to being trans?” The majority (74.9%; *n* = 239) answered affirmatively; 18.2% (*n* = 58) felt there should not be a diagnosis related to being trans; and 6.9% (*n* = 22) had no opinion. The majority of UK HCPs (91.7%; n = 22) thought there should be a diagnosis.

The second question was: “Do you think having a psychiatric diagnosis for gender incongruence (gender dysphoria/gender identity disorder) could have a beneficial effect for adults and adolescents?” Most UK participants (67.7%; *n* = 216) thought that having a psychiatric diagnosis of GI could have beneficial effects for adolescents/adults; 16.0% (*n* = 51) thought that it would not; 5.3% (*n* = 17) had no opinion; and 11.0% (*n* = 35) responded with ‘other, please specify’. Similar results were found from the UK HCPs: Most thought there were benefits (70.8%; n = 17); 25.0% (n = 6) did not; and 4.2% (n = 1) had no opinion.

Furthermore, UK participants were asked: “How would you respond if the adolescent/adult diagnosis for gender incongruence were to be taken out of the chapter on Psychiatric disorders?” UK TG participants most frequently reported that a diagnosis of GI should be removed from the ICD in its entirety (47.0%; *n* = 150); 35.1% (*n* = 112) thought the diagnosis of GI should be allowed to remain in the ICD; 16.9% (*n* = 54) had no opinion; and 0.9% (*n* = 3) said it did not matter. The UK HCPs were split with 45.8% (n = 11) who thought the diagnoses should be retained and 41.7% (*n* = 10) who thought the diagnosis should be removed. The rest had no opinion (8.3%; *n* = 2) or said it did not matter (4.2%; *n* = 1).

### Stigmatization due to a Diagnosis

In the NL survey only, the following question was asked: “Do you consider that having a psychiatric diagnosis (including diagnoses other than gender incongruence) has a stigmatizing effect?” The majority of the participants responded with ‘yes’ (77.8%; *n* = 151); although 9.8% (*n* = 19) of respondents said ‘no’. The remaining participants had no opinion (9.3%; *n* = 18); or gave another response (3.1%; *n* = 6).

In both countries the following question was asked: “Do you consider that having a diagnosis for gender incongruence specifically had a stigmatising effect for adults and adolescents?” Looking at the responses of the Dutch speaking population only—in order to make comparison with the above question—42.3% (*n* = 82) of the respondents answered affirmatively; 25.8% (*n* = 50) said ‘no’; 21.1% (*n* = 41) had no opinion; and 10.8% (*n* = 21) gave another response. This indicates that in the Netherlands and Flanders (Belgium) less participants considered the diagnosis for gender incongruence to be stigmatizing when compared to a psychiatric diagnosis. The question about the gender incongruence diagnosis, did not specify in which chapter that diagnosis would be placed in.

When looking at the TG responses to the above question in both countries, most participants (60.8%; *n* = 312) considered that having a diagnosis for gender incongruence had a stigmatizing effect for adults and adolescents. The study found a statistical difference between the countries regarding this question (*χ*2(3) = 57.82, *p* < .001, Cramer’s *V* = .34). In NL 42.3%, and in the UK 72.1%, considered a diagnosis for gender incongruence to have a stigmatizing effect. Similar results were found for HCPs; most (62.4%) thought a GI diagnosis was stigmatizing for adults and adolescents (see Table 4 below).

**Table 4:** Frequency Table (and Percentage of Column) Regarding Statements About the Stigmatising Effect and/or Recognition as a Result of Having a (Gender Incongruence) Diagnosis.

Question	Possible answer	Country of data collection		
		NL	UK	Total
Do you think having a diagnosis for gender incongruence has a stigmatising effect for adults and adolescents?	No	50 (25.8%)	38 (11.9%)	88 (17.2%)
	Yes	82 (42.3%)	230 (72.1%)	312 (60.8%)
	No opinion	41 (21.1%)	17 (5.3%)	58 (11.3%)
	Other	21 (10.8%)	34 (10.7%)	55 (10.7%)
		*n* = 194	*n* = 319	*n* = 513
Do you think having any kind of psychiatric diagnosis (includingdiagnoses other than gender diagnoses) affords recognition?^a^	No	13 (7.0%)	99 (31.0%)	112 (22.2%)
	Yes	120 (64.5%)	117 (36.7%)	237 (46.9%)
	No opinion	40 (21.5%)	58 (18.2%)	98 (19.4%)
	Other	13 (7.0%)	45 (14.1%)	58 (11.5%)
		*n* = 186	*n* = 319	*n* = 505
Do you think having a diagnosis for gender incongruence affords recognition? ^b^	No	13 (7.0%)	79 (24.8%)	92 (18.2%)
	Yes	129 (69.4%)	165 (51.7%)	294 (58.2%)
	No opinion	21 (11.3%)	37 (11.6%)	58 (11.5%)
	Other	23 (12.4%)	38 (11.9%)	61 (12.1%)
		*n* = 186	*n* = 319	*n* = 505

^a^ The wording for this question in the UK differed slightly: “Do you think having **any kind** psychiatric diagnosis (including diagnoses other than gender diagnoses) affords validation of your identity, practice or issues?”

^b^ The wording for this question in the UK differed slightly: “Do you think having a diagnosis for gender incongruence (gender dysphoria/gender identity disorder) affords validation of your identity, practice or issues?”

When asked if they thought that having *any* psychiatric disorder affords recognition, 46.9% of the participants agreed although 22.2% of the participants disagreed. UK participants differed from NL participants (*χ*2*(3)* = 55.88, *p* < .001, Cramer’s *V* = .33). In the Netherlands 64.5% thought any psychiatric diagnosis could afford recognition, compared to only 36.7% in the UK. In the UK, 31.0%, and in the NL sample 7.0%, thought a psychiatric diagnosis could *not* afford recognition. However, the wording of the question differed between the countries. In the Netherlands the question was: “Do you think any kind of psychiatric diagnosis (including diagnosis other than gender diagnoses) affords recognition” and in the UK: “Do you think having **any kind** psychiatric diagnosis (including diagnoses other than gender diagnoses) affords validation of your identity, practice or issues?” The differences found in responses might be caused by the different wording and interpretation of the questions.

When asked specifically if a gender identity diagnosis could afford recognition (similar to the previous question, the wording differed between the NL and UK), the majority of the respondents (58.2%) agreed but 18.2% of the respondents disagreed. Again, the survey found differences between the UK and the Netherlands (*χ*2(3) = 26.68, *p* < .001, Cramer’s *V* = .23). Of the Dutch participants, 69.4% agreed with the statement that a GI diagnosis can afford recognition whereas in the UK, 51.7% of participants did. UK participants (24.8%) disagreed more frequently than Dutch participants (7.0%) (see Table 4). Similar results were found for HCPs: most (71.7%) thought a GI diagnosis affords recognition.

In summary, most participants considered having a diagnosis for gender incongruence to be stigmatizing for adults and adolescents. Compared to the Dutch participants, UK participants were more concerned about the stigmatization of this diagnosis and were less inclined to think that a GI diagnosis could afford recognition.

### Experiences with Discrimination

The majority of TG respondents (60.4%; *n* = 93 in NL and 84.6%; *n* = 252 in UK) reported having experienced discrimination. When participants were asked to provide examples of discrimination, some of the replies were “*Most days*, *at least something*” (UK participant) or “*Too much to mention*. *I could write a thick book about it*” (Dutch participant).

In specific reports of discrimination, participants mentioned occupational discrimination—some reported losing their employment after starting their transition and others reported not receiving a promotion that they felt was justified. For example, a Dutch participant reported: “*I was rejected for a promotion at work*, *because this would be a ‘difficult situation for others’*.” Some participants reported examples of educational discrimination, for example, as one UK participant noted: “*I was refused a place on a college course because it would 'be too much work*'.”

Other participants reported running into difficulties due to the legal gender documentation that did not match their gender identity and/or presentation; as one Dutch participant reported: “*That my ID card still said I was male*, *while I didn’t look like one*. *Once I was removed from a train by a conductor*, *because I couldn’t prove my identity; mister conductor didn’t believe that it was me on my ID-card*. *But this problem is solved with the new law*.*”* A few participants (in both countries) also reported having been the victim of street-harassment, and/or (sexual) violence. For example, a UK participant reported: “*I have been beaten*, *spat on*, *attacked with a knife*, *sexually assaulted*, *refused access to premises such as shops and gyms*.*”*

Participants reported having experienced discrimination for several reasons (see Table 5). There was a statistical difference between the levels of discrimination reported between countries. In the Dutch speaking countries, 39.6% of participants reported never having been discriminated against, while this was only reported by 15.4% of the UK participants (*χ*2*(1)* = 32.84, *p* < .001, Cramer’s *V* = .27).

**Table 5:** Number of Times a Reason was Ticked (and Percentage of Participants that Selected the Reason) in the Dutch (NL) and United Kingdom (UK) Survey in Response to the Question:: “Have You Ever Been Discriminated Against for any of the Following Reasons:”.

Possible reasons^a^	Country of data collection		
	NL(*n* = 154)	UK(*n* = 298)	Total(*n* = 452)
Gender variant behaviour	54 (35.1%)	170 (57.0%)	224 (49.6%)
Gender dysphoric feelings	36 (23.4%)	140 (47.0%)	176 (38.9%)
The way in which you express your gender identity by your dress or hair style (your gender expression)	70 (45.5%)	197 (66.1%)	267 (59.1%)
Your gender diagnosis	19 (12.3%)	84 (28.2%)	103 (22.8%)
Other reason	15 (9.7%)	5 (1.7%)	20 (4.4%)
I have never been discriminated against	61 (39.6%)	46 (15.4%)	107 (23.7%)

^a^ The percentages indicate the proportion of respondents who selected that reason. Selecting multiple reasons was possible, therefore the sum of the percentages is over 100%.

While many UK participants reported experiencing discrimination, the majority of them (68.5%; *n* = 198) also reported having experienced/experiencing benefits or positives from being a trans person. Examples of benefits or positives include: “*Being able to express myself as me*, *rather than having to stick to gender roles imposed by family/society*” and “*Greater knowledge and understanding about social justice issues*, *particularly sexism*. *Strong companionships with other trans people*. *A deepened relationship with God*.” However 31.5% (*n* = 91) reported not having experienced/ experiencing any benefits or positives and some of the explanations provided by respondents in this group include: “*Stigma*. *It's tough to be visibly trans*” and “*All it does is make life much harder*”.

### Widening the scope of the diagnosis

To find out what participants thought about the broadened diagnostic criteria, the survey asked whether they would consider it an improvement if the scope of the diagnosis was widened. See Table 6 for the responses selected by participants in the UK and NL. The most selected response in both countries was: “”Yes, treatment will thus become available to a more diverse and larger group”, see Table 6. Since the response options differed across the countries, direct comparisons between countries cannot be made.

**Table 6:** Number of Times a Response was given (and Percentage of Participants that Selected the Response Option) in the Dutch (NL) and United Kingdom (UK) Survey Regarding the Statement: ‘Would You Consider it an Improvement if the Scope of the Diagnosis were Widened?’

Possible response^a^	Country of response		
	NL(*n* = 182)	UK(*n* = 317)	Total
No, the risk is too high that people will get the diagnosis even though they don’t need any help: I think it’s stigmatising.	9 (4.9%)	20 (6.3%)	29
Yes, but there is a danger that too many people will get the diagnosis; even those who do not need it. That is stigmatising.	62 (34.1%)	49 (15.5%)	111
Yes, treatment will thus become available to a more diverse and larger group.	99 (54.4%)	174 (54.9%)	273
No opinion	18 (9.9%)	23 (7.3%)	41
Other (NL only)	18 (9.9%)	-	-
No, because (UK only)	-	17 (5.4%)	-
Yes, because (UK only)	-	35 (11.0%)	-

^a^ The percentages indicate the proportion of respondents who selected that reason. Selecting multiple reasons was possible, therefore the sum of the percentages is over 100%.

Regarding the widening of the diagnosis, two options were given: 1) the risk of too many people getting a diagnosis, and 2) the risk of too few people getting a diagnosis. When asked to indicate which of the two risks they would choose if they had to, the survey found that most participants preferred the first option (71.4%; *n* = 130 in NL; 60.5%; *n* = 193 in UK). In the NL 28.6% (*n* = 52) and in the UK 16.9% (*n* = 54) of participants selected the second option. In the UK survey, an extra response option was included: “I don’t know”. This response was selected by 22.6% (*n* = 72) of UK participants. Since the response options differed across the countries, direct comparisons between countries cannot be made. In line with the findings of the TG participants, just over half the Dutch HCPs also preferred the risk of too many people getting a diagnosis over the risk over too few people getting a diagnosis (63.5%; *n* = 47). UK HCPs, on the other hand, selected option 2 most frequently (45.8%, *n* = 11), followed by option 1 (29.2%; *n* = 7).

One of the possible effects of the widening of the diagnosis is that more individuals may apply for treatment, including people with non-binary gender identities. When asked: “Do you think that, if the scope of the diagnosis is widened, more people who do not experience themselves to be either men or women; or who experience themselves to be outside the boxes of manhood or womanhood, etc., will seek care or treatment?”, participants responded with: “I think so, but I'm not sure” (45.7%), followed by “Definitely” (26.9%), see Table 7. Responses differed between the countries (*χ*2(3) = 28.28, *p* < .001, Cramer’s *V* = .24) with the UK being more sure this group would seek treatment in such circumstances than the Netherlands (see Table 7).

**Table 7:** Responses to the Question: “Do You Think That, if the Scope of the Diagnosis is Widened, More People Who Do Not Experience Themselves to be Either Men or Women; or Who Experience Themselves to be Outside the Boxes of Manhood or Womanhood, etc., Will Seek Care or Treatment?”

Possible response	Country of response		Total (*n* = 501)
	NL (*n* = 182)	UK (*n* = 319)	
I don't know	28 (15.4%)	45 (14.1%)	73 (14.6%)
I don't think so	35 (19.2%)	29 (9.1%)	64 (12.8%)
I think so, but I'm not sure	93 (51.1%)	136 (42.6%)	229 (45.7%)
Definitely	26 (14.3%)	109 (34.2%)	135 (26.9%)

### Leaving out the Criterion of Distress and Impairment

When asked if participants thought it was an improvement that the criterion of distress was no longer required, participants in both countries most frequently (59.9% in NL and 48.6% in UK) selected the response option: “Yes, because, this way, people who do *not experience distress* from their gender incongruence but who *do* wish to receive treatment will be able to get a diagnosis and become eligible for treatment,” see Table 8 for all responses.

**Table 8:** Number of Times a Response was given (and Percentage of Participants that Selected the Response Option) in the Dutch (NL) and United Kingdom (UK) Survey Regarding the Statement: ‘In the ICD-11 it is Not Necessary to Experience Significant Distress that Impairs Their Ability to Live a Functional Life. Do You Think That Would be an Improvement?’.

Possible response^a^	*Country of response*		Total
	NL(*n* = 177)	UK(*n* = 319)	
No, because, this way, the diagnosis will also apply to people who experience gender incongruence but don’t have a problem with it. This stigmatises a large group of people.	25 (14.1%)	16 (5.0%)	41
No, because, without experiencing distress, a person should not be able to get a diagnosis.	16 (9.0%)	31 (9.7%)	47
Yes, because, this way, people who do *not experience distress* from their gender incongruence but who *do* wish to receive treatment will be able to get a diagnosis and become eligible for treatment.	106 (59.9%)	155 (48.6%)	261
Yes, because leaving out the criterion of psychological distress means that the diagnosis can be taken out of the psychiatric chapter.	48 (27.1%)	61 (19.1%)	109
No opinion	16 (9.0%)	8 (2.5%)	24
Doesn’t matter	1 (0.5%)	1 (0.3%)	2
Other	25 (14.1%)	11 (3.4%)	36
No because (UK Only)	-	8 (2.5%)	-
Yes because (UK only)	-	28 (8.8%)	-

^a^ The percentages indicate the proportion of respondents who selected that reason. In the Dutch sample, selecting multiple responses was possible (therefore the sum of the percentages is over 100%), in the UK sample it was not.

### Healthcare Funding

Less than half of the participants (42.8%; *n* = 214) expected that there will be changes in healthcare funding for a gender diagnosis if the diagnosis changes; however 34.4% (*n* = 172) of the respondents had no opinion regarding this; and 22.8% (*n* = 114) of the respondents did not think there would be changes in healthcare funding. There was a statistical difference between the responses between the countries (*χ*2(2) = 23.58, *p* < .001, Cramer’s *V* = .22): 26.6% of UK participants (*n* = 85) and 48.1% of Dutch participants (*n* = 87) did not have an opinion; 33.1% of the Dutch participants (*n* = 60) and 48.3% of UK participants (*n* = 154) expected that the healthcare funding will change; and 18.8% of Dutch participants (*n* = 34) and 25.1% of UK participants (*n* = 80) did not expect that healthcare funding would change. Similar results were found for HCPs: around half of the participants (51.0%; *n* = 50) expected that healthcare funding will change if the diagnosis changes.

### Duration of Gender Incongruence

In the proposed diagnosis for adolescents and adults, it will be a requirement for gender incongruence to have lasted for *a few months*. This is to prevent people with highly fluctuating or very recent gender incongruence from getting a premature diagnosis and potentially starting irreversible treatments. In the previous ICD version, a period of two years was applied. Around half of the participants (55.1% in the NL and 48.6% in the UK) thought that the proposed duration of a few months was too short. A small percentage (4.5% in the NL and 13.2% in UK) thought that a few months was still too long (see Table 9). There was a statistically significant difference between the countries (*χ*2(3) = 10.70, *p* = .01, Cramer’s *V* = .15). In line with the transgender respondents, most HCPs (79.8%; *n* = 75) thought the proposed duration of gender incongruence was too short.

**Table 9:** Frequency Table (and Percentage of Column) of Responses to the Question: How do You Feel About Limiting the Duration of Gender Incongruence to a Few Months?

	Country of response		
Possible response	NL(*n* = 176)	UK(*n* = 319)	Total(*n* = 495)
Too short	97 (55.1%)	155 (48.6%)	252 (50.9%)
Still too long	8 (4.5%)	42 (13.2%)	50 (10.1%)
No opinion	25 (14.2%)	53 (16.6%)	78 (15.8%)
Other	46 (26.1%)	69 (21.6%)	115 (23.2%)

The UK survey asked TG participants what they thought was an appropriate time period. There were four possible options: 0–6 months, minimum of 1 year, minimum of 2 years, or 5+ years. Up to 6 months (*n* = 139; 43.6%) and a minimum of 1 year (*n* = 139; 43.6%) were the most frequently chosen options by participants. A total of 36 respondents selected a minimum of 2 years (11.3%) and only 5 selected 5+ years (1.6%). Of the UK HCPs, 16 (66.7%) selected a period of a minimum of a year, 5 (20.8%) selected a minimum of 2 years, 2 (8.3%) selected 0–6 months, and 1 (4.2%) selected 5+ years.

### Disorders/Differences of Sex Development (DSD)-Specification

Most participants in both countries (75.6% in the NL, 90.3% in the UK) thought that individuals with DSD should be able to receive a GI diagnosis. However, in NL 6.8% (*n* = 12) and in the UK 0.9% (*n* = 3) of the participants thought individuals with DSD should not be able to receive a GI diagnosis. Since the response options differed across the countries (in the UK there was a “Other”-option that was not present in the Dutch survey), direct comparisons between countries cannot be made. Similarly, most HCPs (71.0%) thought that individuals with DSD should be able to receive a GI diagnosis.

### Results from the Healthcare Provider Only Questions

Healthcare providers received questions with regard to the consequences of the changes in the criteria for their clinical practice/work which were not of direct relevance to trans people and their family/partners who therefore did not receive this set of questions.

#### Widening the scope of the diagnosis

Healthcare providers were asked if they already met with clients/patients in their work to whom the wider scope of diagnosis might apply (for instance, a person who does not feel to be either man or woman, and who wishes to live their live as a gender-neutral person). Half of the HCPs responded with “yes, sometimes” (*n* = 45; 51.1%). The responses differed between the countries (Fisher’s Exact Test was significant, *p* < .01). Furthermore, the responses differed between HCPs specialized in transgender care and non-specialized HCPs (Fisher’s Exact Test was significant, *p* < .001). See Table 10 for a summary of the results.

**Table 10:** Frequency Table (and Percentage of Column) Regarding the Question (only for Healthcare Providers): ‘Have you Already Met with Clients/Patients in your Work to Whom This Wider Scope of Diagnosis Might Apply (for Instance, a Person Who Does not Feel Themselves to be Either Man or Woman, and Wish to Live Their Live as gender-Neutral, Non-Binary Persons)?’

Possible response	Total(*n* = 88)	Country of response		Specialization	
		NL(*n* = 68)	UK(*n* = 20)	Specialized(*n* = 51)	Not Specialized(*n* = 37)
No, never	23 (26.1%)	23 (33.8%)	0 (0.0%)	5 (9.8%)	18 (48.6%)
Yes, sometimes	45 (51.1%)	29 (42.6%)	16 (80.0%)	29 (56.9%)	16 (43.2%)
Yes, regularly	16 (18.2%)	12 (17.6%)	4 (20.0%)	15 (29.4%)	1 (2.7%)
Other	4 (4.5%)	4 (5.9%)	0 (0.0%)	2 (3.9%)	2 (5.4%)

#### Clarity and applicability of the diagnosis

The majority of healthcare providers (86.7%; *n* = 65) thought the diagnostic criteria were defined clearly; five people (6.7%) thought that the GIAA diagnosis was unclear and five (6.7%) had no opinion. Of these five health care providers (4 specialized, 1 not specialized; 4 from the UK, and 1 from NL)—one thought the wording was too general, one thought the wording was too specific, one had no opinion, and two felt something else about the criteria. One HCP wrote: “*the time limit is random*” and another wrote: “*Trying to fit several people into one name*. *It should be some categories*”.

Most HCPs (70.7%; *n* = 53) thought that the criteria for adolescents/adults would be easy to use in their clinical practice/work; 5.3% (*n* = 4) thought the criteria would not be easy to use; and 24.0% (*n* = 18) had no opinion. There were no differences between countries nor for the level of specialization (Fisher’s exact tests, *p* > .05).

Of the HCPs, 45.9% (n = 34) thought that the duration (several months) was difficult to determine, while 36.5% (*n* = 27) thought it was not difficult. The rest had no opinion (13.5%; *n* = 10); or another response (4.1%; *n* = 3). The responses did not differ for the level of specialization nor between the countries (Fisher’s exact test > .05).

Of the healthcare providers, 40.5% (*n* = 30) thought it was possible to make a distinction between mild GI and a situation in which the diagnosis is met; others thought this was not possible (18.9%; *n* = 14); did not know (27.0%; *n* = 20); or had no opinion (13.5%; *n* = 10). The responses differed between countries (Fisher’s exact test, *p* < .01). In the UK, 55.0% (*n* = 11) of the healthcare providers thought it was possible to make a distinction between mild GI and a situation in which the diagnosis is met; 30.0% (*n* = 6) thought this was not possible; and 15.0% (*n* = 3) had no opinion. No HCP selected “don’t know”. In the NL, 35.2% (*n* = 19) of the healthcare providers thought it was possible to make a distinction between mild GI and a situation in which the diagnosis is met; 14.8% (*n* = 8) thought this was not possible; 13.0% (*n* = 7) had no opinion; and 37.0% (*n* = 20) selected “don’t know”. There were no differences between specialized and not specialized HCPs (*χ*2(3) = 5.28, *p* = .16).

Healthcare providers mostly thought that the removal of the distress criterion would not make it more difficult to diagnose people (48.6%; *n* = 36); however others thought it would become more difficult (31.1%; *n* = 23); and the rest did not know (10.8%; *n* = 8); or had no opinion (9.5%; *n* = 7). There were no differences between countries or level of specialization.

## Discussion

The main aim of this study was to collect the views of service users of transgender healthcare and other stakeholders (i.e., parents of trans children, and siblings or partners of trans people) and healthcare providers regarding the proposed terminology and diagnostic criteria for Gender Incongruence of adolescents and adults in the new edition of the ICD (ICD-11). The study found that overall the proposed changes in the ICD-11 diagnosis of Gender Incongruence of Adolescence and Adulthood were considered an improvement on the ICD-10 diagnosis of Transsexualism. Though a variety of diagnostic terms were mentioned, UK respondents most often chose gender incongruence as the preferred diagnostic term. The two most preferred positions of the GIAA diagnosis in ICD-11 were for it to be placed in a separate chapter dealing with Sexual- and Gender-related Health, or as a Z-code. It should be noted that the name of the new chapter was slightly different in the survey than in the original proposal of the WGSDSH, whose first preference was for placement in a separate chapter focused on gender incongruence only [3]. Their second preference was for the GIAA to be placed in a new chapter focusing on sexual and gender health [3]. In the current ICD beta version, the focus on gender is lost and reads: Conditions related to Sexual Health, see [11]. The preference for placement differed by country; with the first option being preferred by the Dutch and Flemish participants and the latter by the UK participants. This difference may be explained by several factors. First, in the UK, the National Healthcare System (NHS) is funded through central taxation and provides a comprehensive range of health services—the vast majority of which are free at the point of use for people legally resident in the United Kingdom. It is not an insurance-based system and although ICD diagnoses are collected, the funding is not depended upon them; therefore diagnoses which are part of Z-code may still be funded. In contrast, in the Netherlands treatments based on Z-codes are not reimbursed by insurance companies which would make this a less desirable option in terms of service provision. Gender Incongruence as a Z-code might compromise access to care for transgender people, and therefore this placement option may be a less popular choice in the Netherlands than in the UK, where access to care would be unaffected by this change in placement. Second, in the UK, transgender participants more often report experiencing discrimination than in the Netherlands. Also, compared to Dutch participants, more UK participants think a GI diagnosis has a stigmatizing effect. Therefore, the need for destigmatization (in this case through less stigmatizing Z-codes) might be more relevant and stronger in the UK than in NL.

When the ICD-11 proposal was made available—and the survey created—this was based on the assumption that a GIAA diagnosis was retained in ICD-11 and removal of the diagnosis was not recommended because this “would undoubtedly prove to be a significant impediment for transgender people seeking access to medical treatment” (p. 575, [3]). Therefore, questions regarding the possibility of excluding the GIAA diagnosis in its entirety from ICD-11 were not extensively explored in the survey. When asked in which chapter the GIAA diagnosis should be placed in ICD-11, only a small number of participants stated that the GI diagnosis should not be in the ICD at all. This response option was the eighth option out of nine and the question does not necessarily imply that there would be a response option for *not* being in the ICD; so some people might have chosen a category that they found acceptable and might not have finished reading all the options. In other words, their responses might have been based on the idea that if the diagnosis is retained, where should it be placed. The number of people that favored removing the GI diagnosis thus might be higher than the percentage of respondents that picked “It should not be in the ICD at all”.

The UK survey included more questions regarding exclusion of the GIAA diagnosis. The findings on this issue, however, seem to contradict responses to other questions: on the one hand, most UK transgender respondents thought there should be a diagnosis related to being trans and thought there were benefits towards a diagnosis; on the other hand almost half of the transgender respondents indicated they wanted to remove the diagnoses from the ICD in its entirety if it were removed from the chapter on “psychiatric disorders” (healthcare providers were split on this question; some wanted to retain the diagnosis, others preferred removal). Also, when asked about the preferred chapter, only a small number of participants thought the diagnosis should not be in the ICD at all.

As described above, the desire to destigmatize gender incongruence and the importance of securing access to care has been described as the central dilemma in both the DSM-5 and ICD-11 revision processes [3]. This dilemma is clearly reflected in the results of this study. While many participants thought a GI diagnosis has a stigmatizing effect, many also see benefits in having an ICD-11 GIAA diagnosis. The contradicting findings in the UK survey regarding the preference of retaining or removing the GIAA diagnosis (see above) reflects this dilemma. Therefore, at one point in the survey the desire of a respondent to reduce stigma might have been more salient, whereas at other times the benefits of including a diagnosis (e.g., securing access to reimbursed healthcare) may have been more so. It is worth noting that the survey was somewhat lengthy and so shifts in saliency of competing issues and/or desires are likely.

There were no large differences between the responses of the transgender participants and the healthcare providers. Their responses reflected the same dilemmas as the responses of the entire group. Healthcare providers were generally positive about the GIAA diagnosis and most thought the diagnosis was clearly defined and easy to use in their practice/work. The main point of critique of HCPs was aimed towards the duration requirement of “several months”. Almost half of the HCPs thought the duration of “several months” was too short and difficult to determine. Including more practical suggestions by the WHO in the ICD-11 on this matter would be advisable. One such advice could be that a clinician should judge if the gender incongruence is stable enough to permit treatment without mentioning a specific time-frame. Though this may be sufficiently practical for experienced practitioners, it may pose problems for less experienced practitioners.

Some limitations were present in this study. First, the recruitment of the transgender participants started with individuals who intended to receive, are receiving, or have received gender identity care (see Table A in S3 Text). Although this allows comparison between both countries, as it is a relatively homogenous group, it does not allow for the views of non-treatment seeking trans people to be collected. Transgender people who do not want or need medical or psychological care [15] may have a different perspective on the GI diagnosis in the ICD-11; and particularly in the matter of widening the scope of the diagnosis as they will then fulfill diagnostic criteria under the new ICD. Transgender people who do not need/want treatment, but do fulfill the diagnostic criteria, might be more opposed to including any GI diagnosis in the ICD-11 than the group included in this study. For the latter, reimbursed medical treatment is only possible in the NL through a diagnostic code other than a Z-code and in the UK *some* diagnosis must be made for treatment. Therefore, people who need medical care clearly benefit from the presence of a diagnosis as it makes them eligible for reimbursed healthcare. As a result, they might be more positive and less worried about the possible stigmatizing effect of including a GI diagnosis in ICD-11.

A second limitation was the fact that the survey was long and involved some complex concepts. As a result, the sample may have been biased towards participants with a higher level of education, and those who may have difficulties understanding the survey may have given up. In addition, only those with strong opinions may have been motivated to complete the survey to the end. This opens up the possibility that the numbers of potentially more ambivalent people are not accounted for. In addition, no paper surveys were completed which also indicated that only those participants with access to the internet took part, which may exclude certain demographics.

In conclusion, it seems that respondents were overall in favor of the proposed changes regarding the terminology and diagnostic category of Gender Incongruence of Adolescence and Adulthood (GIAA), albeit with some caveats–especially that clear advice as to the duration of the gender incongruence from the WHO is required. If a GIAA diagnosis is retained, it is important that it is not-stigmatizing, and moving it away from the mental and behavioral chapter will likely help with this. Those who oppose removing (or want to retain) a GIAA diagnosis in ICD-11 seem to have a practical view that in some current health care systems, a diagnosis is needed in order to access healthcare that is reimbursed—and so available to a wide variety of people who require treatment.

## Supporting Information

S1 Text The ICD-11 draft criteria of ‘Gender Incongruence of Adolescence and Adulthood’ of the WGSDSH criteria used in this study. (DOCX)

S2 Text The UK Survey (this article reports on the questions marked in yellow). (DOCX)

S3 Text Additional information on the background of the respondents. (DOCX)
