# Supplementary material for: Gender Incongruence of Adolescence and Adulthood: Acceptability and Clinical Utility of the World Health Organization’s Proposed ICD-11 Criteria
Source: PLoS One. 2016 Oct 24;11(10):e0160066. doi: 10.1371/journal.pone.0160066 (PMC5077108; doi:10.1371/journal.pone.0160066)
Supplement: S1 Text — (DOCX) [file pone.0160066.s001.docx]

**S1 Text. The ICD-11 draft criteria of ‘Gender Incongruence of Adolescence and Adulthood’ of the WGSDSH criteria used in this study.**

**Definition:**

Gender Incongruence of Adolescence and Adulthood is characterized by a marked and

persistent incongruence between an individual´s experienced gender and the assigned

sex, which often leads to a desire to ‘transition’, in order to live and be accepted as a person of the experienced gender, through hormonal treatment, surgery or other healthcare services to make the individual´s body align, as much as desired and to the extent possible, with the experienced gender. The diagnosis cannot be assigned prior to the onset of puberty.

**Essential (Required) Features:**

- In adolescents and adults, a marked incongruence between the individual´s experienced gender and the assigned sex, as manifested by at least two of the following:
  - A strong dislike or discomfort with the one´s primary and/or secondary sex characteristics (in adolescents, anticipated secondary sex characteristics) due to their incongruity with the experienced gender.
  - A strong desire to get rid of some or all of one´s primary and/or secondary sex characteristics (in adolescents, anticipated secondary sex characteristics) due to their incongruity with the experienced gender.
  - A strong desire to have the primary and/or secondary sex characteristics of the experienced gender.
  - A strong desire to be treated (to live and be accepted) as a person of the

experienced gender.

- The experienced gender incongruence must have been continuously present for at least several months.
