# Supplementary material for: Gender Incongruence of Adolescence and Adulthood: Acceptability and Clinical Utility of the World Health Organization’s Proposed ICD-11 Criteria
Source: PLoS One. 2016 Oct 24;11(10):e0160066. doi: 10.1371/journal.pone.0160066 (PMC5077108; doi:10.1371/journal.pone.0160066)
Supplement: S3 Text — (DOCX) [file pone.0160066.s003.docx]

**S1 Text. Additional information on the background of the respondents.**

Respondents were asked to indicate which background category applied to them. See Table A for a breakdown of selected respondent categories. Selecting more than one response option was possible in the Dutch survey, but not in the UK survey due to a programming error. However, in the UK survey, some participants used the open text box that was presented after the response option “other, please specify…” to write down the various categories that were applicable to them. Two researchers (GW and TB) used these open answers to tick the response categories (single category or multiple categories) that were relevant for the participant. Based on the respondent type categories selected, participants were divided into three categories: healthcare providers (categories 1, 2, 3 and some who ticked “other, namely…”); (relatives/partners of) transgender persons (categories 5, 6, 7, 8, 9, 10, 12); and participants who are both healthcare providers and (relatives/partners of) transgender persons. When participants selected another category (11), they were placed in one of the three categories based on their open answer. There were no respondents who indicated they were exclusively a teacher (category 4) or an academic researcher (13). Most participants were (relatives/partners of) transgender persons (*n =* 522); 89 participants were healthcare providers; and 17 participants were both healthcare providers and transgender (or relatives/partners), see Table A. These three categories were used to analyse the responses on the survey questions.

**Table A. Number and (and Percentages of Times) a Category was Ticked in the Dutch (NL) and United Kingdom (UK) Survey.**

|  |  | Country of response | |  |
| --- | --- | --- | --- | --- |
| Possible participant category |  | NL^a^  (*n* = 292) | UK^b^  (*n* = 336) | Combined  Data^c^  (*n* = 628) |
| 1. A psychological or medical practitioner specialised in transgender care inside a specialised gender centre |  | 30 (9.2%) | 11 (3.1%) | 41 (6.0%) |
| 2. A psychological or medical practitioner specialised in transgender care outside a specialised gender centre |  | 10 (3.1%) | 3 (0.8%) | 13 (1.9%) |
| 3. A psychological or medical practitioner not specialised in transgender care |  | 35 (10.7%) | 9 (2.5%) | 44 (6.5%) |
| 4. A teacher |  | 10 (3.1%) | 0 (0%) | 10 (1.5%) |
| 5. A trans person who intends to receive, who is receiving, or has received gender identity care |  | 175 (53.7%) | 270 (76.5%) | 445 (65.5%) |
| 6. A trans person who is not receiving transgender care, nor intends to receive it |  | 6 (1.8%) | 7 (2.0%) | 13 (1.9%) |
| 7. A trans person’s parent/guardian |  | 33 (10.1%) | 15 (4.2%) | 48 (7.1%) |
| 8. A trans person’s sibling |  | 1 (0.3%) | 0 (0%) | 1 (0.1%) |
| 9. A trans person’s child |  | 0 (0.0%) | 2 (0.6%) | 2 (0.3%) |
| 10. A trans person’s partner |  | 8 (2.5%) | 9 (2.5%) | 17 (2.5%) |
| 11. Other, namely: …. |  | 18 (5.5%) | 14 (4.0%) | 32 (4.7%) |
| 12. A professional who works in an advocacy/support capacity regarding gender identity care *(UK only)* |  | - | 9 (2.5%) | 9 (1.3%) |
| 13. An academic researcher *(UK only)* |  | - | 4 (1.1%) | 4 (0.6%) |

^a^ Participants could select multiple responses in the Dutch survey. The total of categories selected (*n* = 326) is therefore higher than the number of participants (*n* = 292).

^b^ Respondents could select a single response in the UK survey. However, in an open text box specifying the “other” responses, participants sometimes mentioned multiple categories were applicable to them. Participants were then assigned to those categories mentioned. As a result, the total number of categories ticked (*n* = 353) is higher than the number of participants (*n* = 336).

^c^ For the combined data, again, the total number of categories selected (*n* = 679) is higher than the total number of participants (*n* = 628).
